# Noni juice reduces lipid peroxidation–derived DNA adducts in heavy smokers

**DOI:** 10.1002/fsn3.21

**Published:** 2013-01-07

**Authors:** Mian-Ying Wang, Lin Peng, Claude J Jensen, Shixin Deng, Brett J West

**Affiliations:** 1 Department of Pathology, University of Illinois College of Medicine at Rockford1601 Parkview Avenue, Rockford, Illinois; 2 Research and Development, Morinda, Inc.737 East 1180 South, American Fork, Utah

**Keywords:** Antioxidant, DNA adducts, *Morinda citrifolia*, noni

## Abstract

Food plants provide important phytochemicals which help improve or maintain health through various biological activities, including antioxidant effects. Cigarette smoke–induced oxidative stress leads to the formation of lipid hydroperoxides (LOOHs) and their decomposition product malondialdehyde (MDA), both of which cause oxidative damage to DNA. Two hundred forty-five heavy cigarette smokers completed a randomized, double-blind, placebo-controlled clinical trial designed to investigate the effect of noni juice on LOOH- and MDA-DNA adducts in peripheral blood lymphocytes (PBLs). Volunteers drank noni juice or a fruit juice placebo every day for 1 month. DNA adducts were measured by ^32^P postlabeling analysis. Drinking 29.5–118 mL of noni juice significantly reduced adducts by 44.6–57.4%. The placebo, which was devoid of iridoid glycosides, did not significantly influence LOOH- and MDA-DNA adduct levels in current smokers. Noni juice was able to mitigate oxidative damage of DNA in current heavy smokers, an activity associated with the presence of iridoids.

## Introduction

Cigarette smokers continuously inhale thousands of carcinogens and free radicals. It is estimated that about 10^17^ oxidant molecules are present in each puff of cigarette smoke (DHHS 1989). Free radicals are known to cause oxidative damage by increasing polymorphonuclear leukocytes and by inducing lipid peroxidation (Pryor et al. 1983; Wiencke et al. 1995). Free radicals attach to the unsaturated fatty acids on cell membranes, resulting in a chain reaction that generates lipid hydroperoxides (LOOHs). LOOHs decompose to malondialdehyde (MDA). LOOHs, such as 4,5-epoxy-2(E)-decenal and 4-hydroxy-2-nonenal, as well as MDA, are genotoxic via oxidative reactions and lead to the formation of DNA adducts that are involved in the pathogenesis of tobacco smoke–related cancers and cardiovascular disease (Blair 2001; Nair et al. 2007a; Yanbaeva et al. 2007). Oxidative stress and the consequent lipid peroxidation also have potential roles in the pathogenesis of more than 30 other smoking-related diseases, including cerebrovascular disorders, chronic obstructive pulmonary diseases, and diabetes (Banerjee et al. 1998; Fahn et al. 1998; Altuntas et al. 2002).

Chemical carcinogenesis is a multistage process involving initiation, promotion, and progression (Miller and Miller 1981). Initiation, the critical first step in carcinogenesis, requires the binding of a chemical carcinogen to DNA, thereby forming DNA adducts (Phillips et al. 1988). The analysis of DNA adducts occupies an important area in the fundamental understanding of molecular mechanisms of chemical carcinogenesis. Previously, LOOH was found to be a cofactor in the activation of chemical carcinogens by cytochrome P450 (Wang and Liehr 1995a; Godschalk et al. 2002). This indicates that LOOH has dual carcinogenic roles by damaging DNA directly and accelerating the activation of other carcinogens. Thus, LOOH may contribute continuously to the initiation, promotion, and progression of cancer development.

LOOH- and MDA-DNA adducts have been found in various types of tissues in current smokers (Esterbauer 1993; Marnett 1999; Nair et al. 2007b). Peripheral blood lymphocytes (PBLs) are a good surrogate tissue for the examination of DNA adducts in current smokers. PBLs are more readily accessible than other target tissues, and high correlations have been reported between adduct levels measured in PBLs and other organs, such as the lungs and liver (Nesnow et al. 1993). The change in LOOH- and MDA-DNA adduct patterns and levels in the PBLs of current smokers, before and after preventive intervention, are useful biomarkers for evaluating how effective a preventive strategy is in mitigating oxidative damage of DNA (Bartsch and Nair 2005).

Studies of antioxidant micronutrient supplements, such as alpha-tocopherol, retinyl palmitate, and beta-carotene, did not reveal health benefits for smokers and even suggest possible deleterious effects (Omenn et al. 1996; Kelly 2002a; Omenn 2007). This may be due to the fact that high doses of these antioxidant micronutrients may exert pro-oxidant effects under certain conditions, such as smoking (Mayne et al. 1996; Palozza 1998). On the other hand, such adverse effects have not been observed in studies involving foods rich in antioxidants, where various natural forms of these nutrients are present in concentrations less than in the supplements (Kelly 2002b; Gibson et al. 2010). Fruits and vegetables are major sources of dietary antioxidants. Epidemiological studies indicate that fruits and vegetables may reduce free radical–induced oxidative damage and lipid peroxidation in cigarette smokers (Steinmetz and Potter 1991). *Morinda citrifolia* (noni) is an evergreen small tree that grows in many tropical regions of the world. Noni fruit has a significant history of use as both food and medicine among Pacific Islanders and in Southeast Asia (Morton 1992; West et al. 2006). Various potential health benefits of noni fruit have been reported (Wang et al. 2002), including immunomodulation (Hirazumi and Furusawa 1999; Palu et al. 2008a) and antioxidant activities in vitro and in vivo (Wang and Su 2001; Mohd-Zin et al. 2002; Su et al. 2005). Noni juice has been found to exert an antioxidant effect in human athletes, resulting in increased endurance (Palu et al. 2008b). Noni juice also lowered plasma concentrations of superoxide anion radicals (SAR) and LOOHs in heavy smokers (Wang et al. 2009a). Given its demonstrated antioxidant activity, noni juice may also reduce cigarette smoke–induced oxidative damage of DNA. As such, this study was designed to investigate the influence of noni juice on the generation of LOOH- and MDA-DNA adducts in PBLs of current heavy smokers.

## Material and Methods

### Study ethics

The research protocol of this trial was approved by the Institutional Review Board of the University of Illinois College of Medicine at Rockford, following an ethics review. The trial was conducted according to the Declaration of Helsinki, and written informed consent was obtained from all participants.

### Study participants

Adult heavy smokers were recruited for enrollment in this study. The inclusion criteria were 18–65 years in age, smoker of more than 20 cigarettes per day, a smoking history exceeding 1 year, and no concurrent use, or use in the previous 3 months, of prescription medication or antioxidant vitamins. Those enrolled had to be willing to complete a 1-month trial. Those interested in participating were interviewed by an experienced clinical coordinator and selected for participation if they met eligibility criteria. The clinical coordinator interviewed all study participants and asked them to complete a demographic and health information questionnaire. Study participants were randomly assigned to a 118-mL placebo, 29.5-mL noni, or 118-mL noni dose groups. Both males and females were enrolled in equal proportions.

### Noni fruit juice and placebo

The European Union–approved form of noni fruit juice from Tahiti (Tahitian Noni® Juice; Morinda, Inc., Provo, UT) was used for this trial. The placebo consisted of a blend of grape and blueberry juices and natural cheese flavor to mimic the flavor of Tahitian Noni® Juice. It also served as an iridoid deficient fruit juice control.

### Intervention

Participants and investigators were blinded as to which treatment group each volunteer was assigned. Those in the 29.5-mL group were asked to drink the noni juice all at once in the morning and on an empty stomach. Those in the other two groups were asked to drink 59 mL twice daily (118 mL daily total), once in the morning on an empty stomach, and once before bedtime. This schedule was followed for 30 days. Participants were not asked to alter their smoking habits during the intervention period, and an assumption was made that they continued to smoke in the same manner (amount and duration) as they had before enrollment in the trial.

### ^32^P-postlabeling assay

Ten milliliters of whole blood were drawn from each participant upon enrollment and again at completion of the intervention period. Blood samples were drawn into tubes containing heparin, which were then centrifuged at 1500*g* for 20 min to remove plasma. The remaining fractions were transferred to 50-mL tubes. Fifteen milliliters of red blood cell lysis buffer (150 mmol/L NH_4_Cl, 10 mmol/L sodium hydrogen carbonate, 1 mmol/L ethylenediamine tetraacetic acid, pH 7) was added, and the mixture was incubated at room temperature for 5 min. Samples were then centrifuged at 300*g* for 10 min at 4°C. The PBL pellet was washed with 5 mL of red blood cell lysis buffer, incubated at room temperature, and centrifuged at 300*g* for 10 min at 4°C. This procedure was repeated three times. The final PBL pellet was resuspended with 0.5 mL of buffer (150 mmol/L sodium chloride, 10 mmol/L EDTA, pH 8.0), and cells were lysed by vortex. DNA was then isolated with the FastDNA® kit (MP Biomedicals, Solon, OH). DNA samples were stored at −80°C until ^32^P-postlabeling.

DNA adducts were measured by the nuclease P1 procedure of the ^32^P-postlabeling assay (Reddy and Randerath 1986). Five to ten micrograms of DNA were digested with micrococcal endonuclease and spleen phosphodiesterase to produce 3′ mononucleotides. Enrichment of adducts involved dephosphorylation of unmodified normal nucleotides with nuclease P1 to prevent radiolabeling. Adducted nucleotides were then labeled with [γ-^32^P]ATP by T4 polynucleotide kinase (Wang and Liehr 1995a). Labeled nucleotides were purified by spotting nucleotide solutions onto polyethyleneimine-(PEI-)cellulose thin layer chromatography (TLC) plates, followed by removal of normal nucleotides by plate development with a sodium phosphate mobile phase (2.3 mol/L, pH 5.75) and Whatman 1 paper wick (Whatman Ltd., Maidstone, U.K.). Afterward, the chromatogram was cut into 1.0 × 2.4 cm strips beginning at 2.4 cm above the origin point. The central and furthest (upper) strips, which contained LOOH adducts (such as 4-hydroxy-2-nonenal) and MDA adducts, respectively, were retained. Adducts were transferred from each strip to fresh PEI-cellulose TLC sheets by a magnet transfer technique (Lu et al. 1986). Two-dimensional chromatography was completed with different mobile phases. For the first direction, a 3.6 mol/L lithium formate and 8.5 mol/L urea solution (pH 3.6) served as the mobile phase. The plate was then developed in a second direction, at a right angle to the first, with a mobile phase composed of 0.8 mol/L lithium chloride, 0.5 mol/L Tris–HCl, and 8.5 mol/L urea (pH 8.0). Adducts were detected by autoradiography at −80°C using Kodak X-OMAT (Eastman Kodak Co., Rochester, NY) film and DuPont Lightning Plus intensifying screens (DuPont, Wilmington, DE) (Wang and Liehr 1995a). Adduct spots were removed from chromatograms and transferred to scintillation vials. Counts per minute from each sample were measured in a scintillation counter. Adduct levels were expressed as relative adduct labeling (RAL), which is the ratio of counts per minute of adducted nucleotides and counts per minute of total nucleotide samples. A value of 1 RAL × 10^9^ corresponds to 1 adduct in 10^9^ bases (Wang and Lu 1990).

### Statistical analysis and data interpretation

A power analysis was performed to estimate the number of cases needed to detect a significant effect (Brookes et al. 2001). As the study was designed to compare predata and postdata, all analyses were conducted on paired cases in each group. To assess the influence of noni juice and placebo on LOOH- and MDA-DNA adduct levels, the average adduct levels were compared before and after the intervention in each group using a paired Student's *t*-test. Pearson's chi-squared test was used to evaluate intergroup differences.

### Chemical analyses

The iridoid content, inclusive of deacetylasperulosidic acid (DAA) and asperulosidic acid (AA), was determined by HPLC, according to a previously reported method (Deng et al. 2011). Other significant secondary metabolites, such as scopoletin, rutin, and quercetin, were also determined by HPLC.

HPLC grade acetonitrile (MeCN), methanol (MeOH), and water were obtained from Sigma-Aldrich (St. Louis, MO). Analytical grade formic acid was purchased from Spectrum Chemical Mfg. Corp. (New Brunswick, NJ). DAA and AA standards were isolated from authentic noni fruit in our laboratory. Their identification and purities were determined by HPLC, mass spectrometry, and nuclear magnetic resonance to be higher than 99%. They were accurately weighed and then dissolved in an appropriate volume of MeOH to produce corresponding stock solutions. The working standard solutions of DAA and AA for the calibration curve were prepared by diluting stock solutions with MeOH in seven concentration increments ranging from 0.00174–1.74 and 0.0016–0.80 mg/mL, respectively. All stock and working solutions were maintained at 0°C. The calibration curves of the standards were plotted after linear regression of the peak areas versus concentrations.

For iridoid analyses, samples of noni juice and placebo were diluted with MeOH-H_2_O (1:1) and then filtered through a 0.45-μm nylon membrane filter. Chromatographic separation was performed on a Waters 2690 separations module coupled with 996 photodiode array (PDA) detectors, equipped with a C18 column (4.6 × 250 mm, 5 μm; Waters Corporation, Milford, MA). The pump was connected to two mobile phases: (A) MeCN and (B) 0.1% formic acid in H_2_O (v/v), and eluted at a flow rate of 0.8 mL/min. The mobile phase was programmed consecutively in linear gradients as follows: 0–5 min, 0% A; and 40 min, 30% A. The PDA detector was monitored in the range of 210–400 nm. The injection volume was 10 μL for each of the sample solutions. The column temperature was maintained at 25°C. Data collection and integration were performed using Waters Millennium software revision 32 (Waters Corp., Milford, MA).

Analyses of scopoletin, rutin, quercetin, and chlorogenic acid were also performed by HPLC, according to a previously reported method (Deng et al. 2010). Chemical standards were accurately weighed and then dissolved in an appropriate volume of MeOH/MeCN to produce corresponding stock and working standard solutions. Chromatographic separation was performed on a Waters 2690 separations module coupled with a 996 PDA detector, and equipped with a C18 column. The mobile phase system was composed of three solvents: (A) MeCN; (B) MeOH; and (C) 0.1% TFA in H_2_O (v/v). The mobile phase was programmed consecutively in linear gradients as follows: 0 min, 10% A, 10% B, and 80% C; 15 min, 20% A, 20% B, and 60% C; 26 min, 40% A, 40% B, and 20% C; 28–39 min, 50% A, 50% B, and 0% C; and 40–45 min, 10% A, 10% B, and 80% C. The elution was run at a flow rate of 1.0 mL/min. The UV spectra were quantified at 365 nm.

Total polyphenols were determined by the Folin–Ciocalteu method. Samples were centrifuged and diluted 1:10 with deionized water. The diluted samples (10 μL) were mixed with 800 μL deionized water and 50 μL Folin–Ciocalteu (2N). Following incubation at room temperature for a few minutes, 150 μL Na_2_CO_3_ (saturated) was added, and sample tubes were shaken and allowed to incubate at room temperature for 2 h. Vehicle blanks and gallic acid standards were prepared in the same manner. Following incubation, the absorbance of the blanks, standards, and samples were measured at 765 nm in a microplate reader. Absorbance versus gallic acid concentration was used to create a calibration curve. This curve was used to determine the total phenol content of the samples. As noni fruit is a source of vitamin C (West et al. 2011), concentrations of this vitamin in both the placebo and noni juice product were also measured after pasteurization and bottling.

## Results

### Participant demographics

There were no significant differences in demographics between the groups. A 1:1 gender ratio was maintained in each group, with mean age ranging from 37 to 43 years. The average number of cigarettes smoked per day in each group was from 26 to 28.6, and average pack-years (number of cigarettes smoked daily multiplied by years of smoking) ranged from 32.12 to 32.49. The ethnic compositions of each group were primarily Caucasian, 76–95%, and African American, 8–22%. There were no differences in the mean number of cigarettes smoked each day. Of the 317 smokers enrolled, 245 completed the trial. Finally, the 118-mL noni juice group had a larger proportion of missed doses than the 29.5-mL group. The proportion of individuals missing <5 doses in the 29.5-mL and 118-mL noni juice groups were 30% and 32%, respectively. The rate of those missing 5–10 doses in the same low- and high-dose groups were 2% and 10%, respectively.

### Changes in DNA adduct levels

Accumulated LOOH- and MDA-DNA levels are useful biomarkers for determining the efficacy of an antioxidant and chemopreventive intervention. We chose these biomarkers to examine whether noni juice, a proven antioxidant in vitro and in vivo, was able to decrease LOOH- and MDA-DNA adduct levels in current smokers after a 1-month intervention.

**Figure 1:**
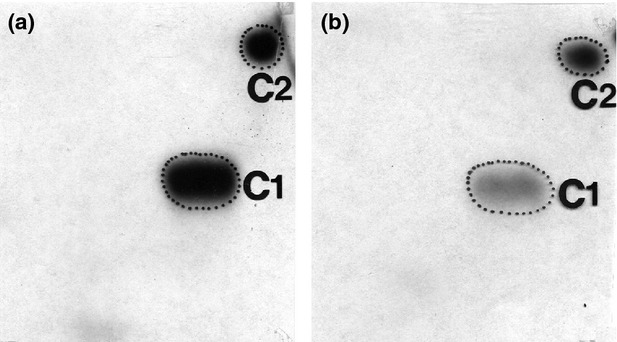
Autoradiograph profiles of LOOH-DNA adducts (C1 and C2) in peripheral blood lymphocytes of current smokers before (a) and after (b) intervention.

**Figure 2:**
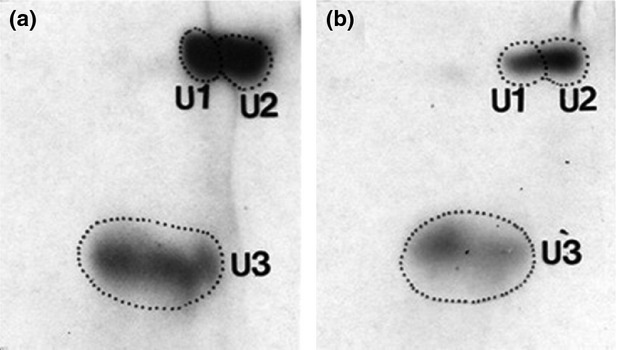
Autoradiograph profiles of malondialdehyde (MDA)-DNA adducts in peripheral blood lymphocytes of current smokers before (a) and after (b) intervention. Spot U3 corresponds to MDA-deoxyguanosine adducts, while U1 and U2 are MDA-deoxyadenosine adducts.

Expected adducts were successfully detected in PBLs of the current smokers, with typical LOOH- and MDA-DNA adduct profiles being present (Wang and Liehr 1995b; Wang et al. 1996). Examples of pre- and postautoradiographs of LOOH-DNA adducts, labeled C1 and C2, from one volunteer are provided in Figure 1. Adducts densities decreased significantly following 4 weeks of daily ingestion of 29.5 mL noni juice. Pre- and postautoradiographs of MDA-DNA adducts (labeled U1, U2, and U3) are provided in Figure 2. Densities of these also decreased significantly with noni juice intake.

Mean pretest and posttest LOOH- and MDA-DNA adduct levels in the placebo and both noni juice dose groups are compared in Table 1. No significant decreases were observed in the placebo group (*P* > 0.05). Noni juice ingestion decreased lipid peroxidation–derived DNA adduct levels in PBLs by approximately half. Percent reductions in LOOH-DNA adducts in the 29.5- and 118-mL noni juice groups were 54.9% (*P* < 0.001) and 44.6% (*P* < 0.001), respectively. Mean posttest MDA-DNA levels in both noni juice groups ranged from 46.9% to 57.4% (*P* < 0.001) lower than pretest values. Posttrial DNA adduct levels in the 118 mL are not significantly lower than the 29.5-mL group. This may indicate a possible threshold of antioxidant activity that is reached by a daily dose of 29.5 mL, but may also be due to lower compliance in the 118-mL noni group. This group had a greater percentage of missed doses than the 29.5-mL group. There was no statistically significant change in adduct levels in the placebo group despite a trend for increased posttrial MDA-DNA adduct levels. In fact, a high degree of variability was evident in pretest and posttest adduct levels of the placebo group. Participants were not asked to comply with a specified diet during the trial or to refrain from alcohol consumption. Therefore, individual differences in dietary and lifestyle habits may produce greater variability given the smaller sample size of the placebo group. Even so, the placebo apparently provided no antioxidant effect strong enough to produce a significant and consistent decline in DNA adducts among study participants.

**Table 1:** Lipid peroxidation–derived DNA adduct levels in peripheral blood lymphocytes of current heavy smokers

	Placebo	29.5 mL noni	118 mL noni
LOOH-DNA adducts (RAL × 10^9^)			
Pretest	169 ± 134	71.3 ± 6.7	62.0 ± 6.9
Posttest	99 ± 100	32.1 ± 2.3^1^	34.3 ± 2.2^1^
MDA-DNA adducts (RAL × 10^9^)			
Pretest	192 ± 26.6	89.3 ± 12.3	77.8 ± 10.7
Posttest	866.2 ± 235.3	38.0 ± 2.2^1^	41.3 ± 2.7^1^

MDA, malondialdehyde; RAL, relative adduct labeling.

1 *P* < 0.0001 compared with pretest values.

**Figure 3:**
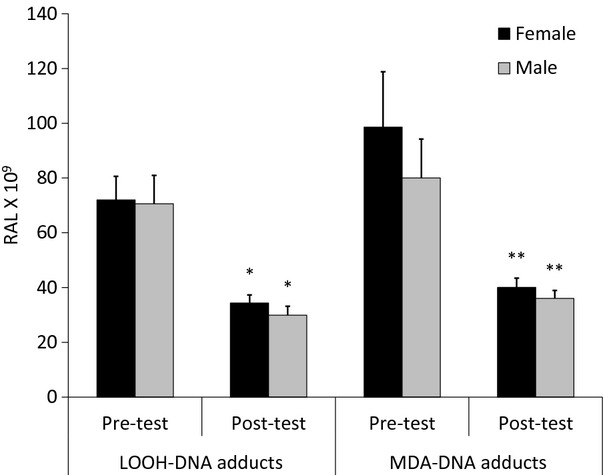
Lipid peroxidation–derived DNA adduct levels, by gender, in the 29.5-mL noni juice dose group. *P < 0.0001 compared with pretest values. **P ≤ 0.002 compared with pretest values.

**Figure 4:**
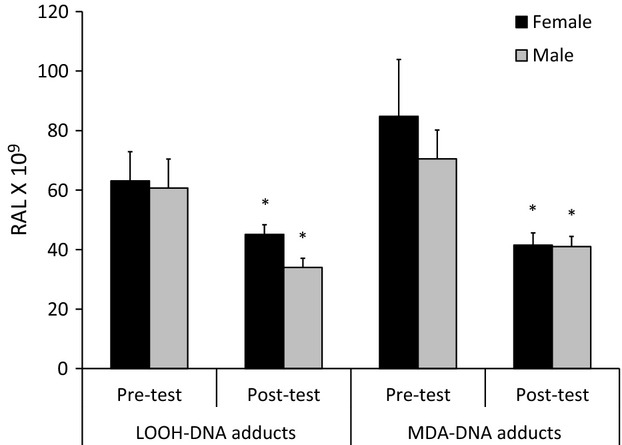
Lipid peroxidation–derived DNA adduct levels, by gender, in the 118-mL noni juice dose group. *P = 0.01 compared with pretest values.

No significant gender differences were evident in average LOOH- and MDA-DNA adduct levels in either noni juice dose group. But intragroup reductions did occur in both females and males (Figs. 3, 4). The approximate halving of adduct levels seen in the nongender-specific comparisons is evident in both males and females in the 29.5-mL group, with 52.4–59.3% reductions in both types of adducts. A 51.1% decline in MDA-DNA adducts also occurred in females of the 118-mL group. However, mean LOOH-DNA adduct values of these females declined by only 45.1%. Males at this dose experienced a 41.8–44.1% decline in both adduct types.

### Adverse events

There were no adverse events, including symptoms of illness or injury, in the placebo or noni juice groups during the intervention period.

### Chemical comparison of noni juice and placebo

The phytochemical compositions of the noni juice product and placebo used in this trial are compared in Table 2. The total polyphenol content of each of these was similar, with no substantial difference in flavonoid (quercetin and rutin) or chlorogenic acid concentrations. Iridoids, which were present in significant quantities in the noni juice product, were absent in the placebo. Scopoletin was also detected in noni juice, but the content was minor compared with the total iridoid concentration and was below the quantity previously demonstrated to provide effective antioxidant action (Panda and Kar 2006; Ding et al. 2008). The vitamin C contents of noni juice and placebo were not significant, with both being <0.2 mg/mL. In this trial, it is apparent that the protection of DNA from oxidative damage caused by cigarette smoke is associated with the presence of iridoids, specifically DAA and AA. This association is consistent with results of in vitro tests with these iridoids (West et al. 2011).

**Table 2:** Phytochemical compositions (mean ± standard deviation) of the noni juice product and placebo evaluated in the clinical trial

Analysis	Noni juice	Placebo
Chlorogenic acid (mg/mL)	0.0831 ± 0.0015	0.1030 ± 0.0020
Rutin (mg/mL)	0.0349 ± 0.0004	0.0252 ± 0.0004
Total polyphenols (mg/mL)	0.6200 ± 0.0400	0.4900 ± 0.0600
Total iridoids	0.5115 ± 0.0162	None detected
Deacetylasperulosidic acid (mg/mL)	0.3747 ± 0.0158	None detected
Asperulosidic acid (mg/mL)	0.1122 ± 0.0070	None detected
Scopoletin (mg/mL)	0.0139 ± 0.0005	None detected

## Discussion

The results of our current trial are consistent with previous reports of noni juice consumption by heavy smokers. For example, mean plasma SAR and LOOH concentrations, other markers of oxidative stress, were lowered significantly by 26.9% and 24.5%, respectively, in heavy smokers after 4 weeks of ingesting 29.5 mL noni juice daily (Wang et al. 2009a). As such, the reduction of MDA and LOOH DNA adducts seen in the current trial is expected as SAR and LOOH lead to the formation of these adducts (De Bont and van Larebeke 2004). The reduction in lipid peroxide–derived DNA adducts levels in this study follows the same trend as for aromatic DNA adducts, as previously reported (Wang et al. 2009b). Therefore, the combined data suggest that noni juice may be able to provide dual protection against downstream oxidative damage of DNA and more direct electrophilic attack by carcinogens in tobacco smoke and their metabolites (Hang 2010).

The apparent antioxidant activity demonstrated in this current trial is consistent with previous in vitro and in vivo antioxidant studies of noni. Mohd-Zin et al. (2002) revealed that the ethyl acetate extract of noni fruit prevented peroxide formation in the ferric thiocyanate assay at the same rate as α-tocopherol and 2,6-di-tert-butyl-p-cresol (BHT), both well-known antioxidants that have been widely used in foods. They also found no significant differences in the efficacies of this extract and these two antioxidants in lowering the amount of thiobarbituric acid reactive substances in vitro. Calzuola et al. (2006) demonstrated that an aqueous ethanol extract of noni fruit juice also possessed strong SAR scavenging activity in the nitrotetrazolium blue chloride (xanthine/xanthine oxidase) assay. The same noni juice product evaluated in this study was previously evaluated for its antioxidant activity in vitro by Wang and Su (2001). They compared the SAR scavenging activity of 7 μL/mL noni juice to those of 13.3 μg/mL vitamin C, 13.3 μg/mL Pycnogenol® (Twinlab Corp., Ronkonkoma, NY), and 22.2 μg/mL grape seed powder. On a per serving basis, the SAR scavenging activity of noni juice compared very favorably to these other antioxidant rich substances. Furthermore, they demonstrated significant LOOH quenching activity of whole noni juice in vitro.

Significant antioxidant activity of noni juice has been reported in an acute liver injury model with carbon tetrachloride (Wang et al. 2001). The pathogenesis of carbon tetrachloride (CCl_4_) includes extensive damage from lipid oxidation processes, due to the formation of the trichloromethyl radical during metabolism. The experiment compared noni juice to the same placebo used in this trial. Female Sprague–Dawley rats were fed 20% noni juice or placebo in drinking water for 12 days. On the final day, animals from the placebo and control groups were administered 0.25 mL/kg CCl_4_ by gavage and then sacrificed 3 h afterward. The livers were removed and the SAR and LOOH levels were measured and compared. The SAR and LOOH levels of the liver tissue from animals pretreated with noni juice were 50% and 20% lower, respectively, than those measured in liver tissues from placebo pretreated animals. The results indicate that noni juice has a protective influence in the presence of oxidative stress in vivo.

In the current trial, the phytochemical analysis of the noni juice product and the placebo indicates that iridoids, specifically DAA and AA, are the major point of difference. Iridoids are known for antioxidant activities (Tundis et al. 2008). Oleuropein, a secoiridoid, is perhaps the best characterized relative to its antioxidant capacity (Vissers et al. 2004; de la Torre-Carbot et al. 2010; Omar 2010). However, data demonstrating the antioxidant potential of iridoid glycosides are also emerging. Two iridoids that are structurally similar to those found in noni fruit are loganic acid and loganin. Loganic acid reduced superoxide generation in human neutrophils activated by *N*-formyl-methionyl-leucyl-phenylalanine and arachidonic acid. Loganic acid inhibited the superoxide generation in a concentration-dependent manner (Wei et al. 2012). Loganin exhibited antioxidant activities in rat renal mesangial cell cultures incubated in the presence of advanced glycation end products, inducers of cellular oxidative stress. Cells incubated with loganin for 48 h exhibited increased antioxidant enzyme activity, such as superoxide dismutase and glutathione peroxidase activities, and decreased MDA concentration (Xu et al. 2006).

Similar to the antioxidant activity of oleuropein in olive, iridoids occurring in noni fruits inhibited the oxidation of low-density lipoproteins (LDL) in vitro. Both DAA and AA demonstrated significant inhibition of copper sulfate–induced oxidation of human LDL (Kim et al. 2005). Also, DAA and AA prevented 4-nitroquinoline 1-oxide (4NQO)–induced DNA damage in vitro (West et al. 2011). 4NQO is a genotoxin that causes the formation of 8-hydroxydeoxyguanosine (8OHdG), a product of DNA oxidation. 4NQO exposure leads to the formation of superoxide, hydrogen peroxide, and hydroxyl radicals, resulting in the production of a substantial amount of 8OHdG in DNA in mammalian and bacterial cells (Nunoshiba and Demple 1993; Arima et al. 2006). Treatment with DAA and AA reduced 4NQO genotoxicity in prokaryotic cells by 98.96% and 99.22%, respectively. Therefore, the in vitro oxidative activity of 4NQO was almost entirely abolished by the addition of either iridoid, further suggesting that iridoids may be responsible for the protective effects of noni juice observed in this trial.

The oxidative damage of DNA does lead to adverse health effects. A meta-analysis found an association between DNA adducts and lung cancer in current smokers (Veglia et al. 2008). However, the oxidative damage of DNA is not limited to tobacco smoke exposure but is also caused by other sources of polycyclic aromatic hydrocarbons (PAHs), which seem to be ubiquitous environmental pollutants (Vineis and Husgafvel-Pursiainen 2005). It is reasonable, therefore, to assume that it is possible that noni juice consumption may provide similar effects in nonsmokers in PAH-polluted environments, and even more so to those exposed to second-hand smoke (Howard et al. 1998). However, further research will be important to understand the magnitude of potential effects in nonsmokers.
